# Identification and Functional Characterization of a Novel Variant in the *SEMA3A* Gene in a Chinese Family with Kallmann Syndrome

**DOI:** 10.1155/2022/2504660

**Published:** 2022-10-11

**Authors:** Meng Shu, Huixiao Wu, Shuoshuo Wei, Yingzhou Shi, Zongyue Li, Yiping Cheng, Li Fang, Chao Xu

**Affiliations:** ^1^Department of Endocrinology and Metabolism, Shandong Provincial Hospital, Shandong University, Jinan, Shandong 250021, China; ^2^Shandong Provincial Key Laboratory of Endocrinology and Lipid Metabolism, Institute of Endocrinology and Metabolism, Shandong Academy of Clinical Medicine, Jinan, Shandong 250021, China

## Abstract

**Background:**

Kallmann syndrome (KS) is a rare genetic disease characterized by the reproductive system and olfactory dysplasia due to the defective migration of gonadotropin-releasing hormone (GnRH) neurons. However, this disorder is clinically heterogeneous and the genotype-phenotype relationship has not been determined.

**Objective:**

The present study aimed to identify the variant causing KS in a Chinese family and evaluate the functional consequences and phenotypes associated with the novel variant.

**Methods:**

A Chinese family with KS was screened for pathogenic variants by whole-exome sequencing (WES). Bioinformatic analysis was performed to predict the consequences of the identified variant. The expression of the mutant protein was examined *in vitro*.

**Results:**

A novel heterozygous variant (NM_006080.2 : c.814G > *T*) in *SEMA3A* was identified in the patient and his father, which caused the substitution of aspartic acid with tyrosine in codon 272. It was predicted to result in pathogenic significance with a high damaging score and seriously affect protein structure by bioinformatic analysis. *In vitro* experiments revealed this variant could significantly decrease the expression of SEMA3A. Furthermore, it may cause the disease by failing to induce the phosphorylation of focal adhesion kinase (FAK) in GnRH neurons.

**Conclusion:**

Identification and functional characterization of this novel variant in the *SEMA3A* gene in a Chinese family with Kallmann syndrome extend the genetic variant spectrum of *SEMA3A* and provide more data about the heterogeneity of KS, which may provide further insights into the diagnosis of KS and help patients get additional data in genetic counseling and timely treatment.

## 1. Introduction

Idiopathic hypogonadotropic hypogonadism (IHH) is a rare genetic disease characterized by delayed or absent puberty and infertility due to an isolated defect in gonadotropin-releasing hormone (GnRH) secretion or action with an incidence of 1 : 10000 in males and 1 : 50000 in females [1–4]. When IHH is accompanied by anosmia or hyposmia, it is named Kallmann syndrome (KS), and when the sense of smell is normal, it is considered normosmic idiopathic hypogonadotropic hypogonadism (nIHH) [5]. KS has obvious clinical and genetic heterogeneity, and the majority of cases are sporadic while the remaining cases are familial [6]. Even in family cases, the clinical phenotypes of the same genetic defect can vary widely among affected family members [5, 7]. To date, IHH is caused by variants in approximately 50 genes, of which about 20 genes can lead to KS [8, 9]. Among these genes, semaphorin 3A (*SEMA3A*), which belongs to the Class 3 semaphorin family, encodes a secreted protein that plays an important role in the development of the nervous system and in axonal guidance [10]. However, *SEMA3A* variants only account for around 6% of KS cases and the related molecular mechanism remains ambiguous, which needs further research [11, 12].

**Figure 1:**
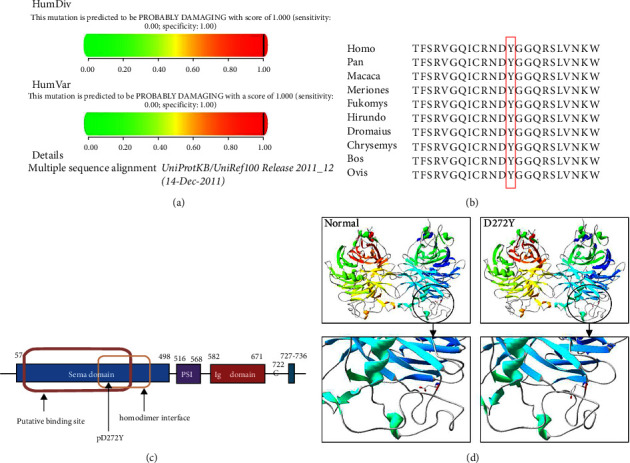
Bioinformatic analysis, domains and protein structure prediction of the novel variant in the *SEMA3A* gene. (a) Score of the novel damaging variant c.814G > *T* (p.D272Y) in Polyphen v.2. (b) Cross-species conservation of SEMA3A around p.D272 is displayed. (c) The structure domains of *SEMA3A.* The arrow below the domains on the left indicates the putative plexin binding region, the right is the homodimer interface where polypeptide binds, and the novel variant site of *SEMA3A* is located at the middle. (d) Protein structure prediction of wild-type and mutant *SEMA3A*.

*SEMA3A* is located at 7q21.11 and encodes SEMA3A with a Sema domain, a plexin/semaphorin/integrin (PSI) domain, an immunoglobulin-like (Ig) domain, and cysteine residue(*C*) (Figure 1(c)). The Sema domain is a distinct protein domain with the highly structurally conserved seven-blade *β*-propeller fold structure, which is present as a single copy located at the N-terminus of SEMA3A [13]. Interestingly, the Sema domain is also present in plexin family proteins and several receptor tyrosine kinases and mediates homophilic dimerization between Semas [14]. In 2011, a study reported that GnRH neuronal migration defects, abnormal olfactory bulb development, and hypogonadism appeared in *SEMA3A* knockout mice [15]. In 2012, Young et al. demonstrated that the heterozygous deletion of 11 out of the 17 *SEMA3A* exons resulted in phenotypes related to KS [11]. Subsequently, Hanchate et al. and Känsäkoski et al. demonstrated that most of the *SEMA3A* variants identified in KS patients were loss of function [12, 16].

Up to now, 46 variants in the *SEMA3A* gene are listed in the HGMD database (https://www.hgmd.org/), of which 18 are associated with KS, and among them, even fewer have undergone functional analysis. The genotype-phenotype relationship is not properly clear, especially when the patient's symptoms are atypical. Thus, it is quite essential to detect more variants and conduct genotype-phenotype analysis. In this study, we identified a novel missense variant in the *SEMA3A* gene in a pedigree with KS by whole-exome sequencing (WES). The results of functional experiments showed that this variant could significantly reduce the expression of SEMA3A and may affect downstream signaling. Meanwhile, we summarized all the reported *SEMA3A* variants and molecular mechanisms based on the present data. Our study expands the genotypic spectrum of *SEMA3A* variants associated with KS and provides additional data to aid in genetic counseling for patients who are suffering from this disorder.

## 2. Subjects and Method

### 2.1. Subjects

The study was conducted in accordance with the Declaration of Helsinki and approved by the institutional review board of Shandong Provincial Hospital. The subject family with KS was enrolled in our study with informed consent in September 2019. Peripheral blood samples were collected from the proband and his parents for genetic analysis. The clinical information, including medical history, physical examination, laboratory examination, and imaging examination, was also collected. A Sniffin' Sticks 16-identification test (SIT-16, GM UK), a smell identification test, was used to evaluate their olfactory function.

### 2.2. Pedigree

**Figure 2:**
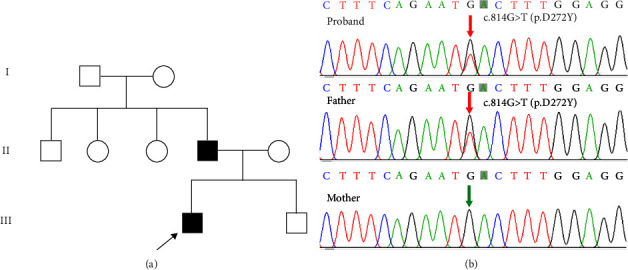
The Chinese family with KS in our study. (a) Pedigree of the family with SEMA3A variant. Squares: males; circles: females; filled symbol: affected; unfilled symbol: unaffected; arrows: proband. (b) Partial DNA sequence diagram of SEMA3A in our case. A novel heterozygous variant (c.814G > *T*) in SEMA3A of II-4 and III-1 was identified by WES, causing the substitution of aspartic acid with tyrosine in codon 272, as shown by an arrow.

The pedigree, a three-generation family, consisted of 9 individuals and was identified in Jinan, Shandong Provincial, China (Figure 2(a)). The proband, a 31-year-old married man, was referred to the Shandong Provincial Hospital complaining of infertility. His father had been identified with Kallmann Syndrome before. His parents were nonconsanguineous. Other members of the family were all phenotypically healthy at the time of the study.

### 2.3. Sequencing and Bioinformatics

Genomic DNA was extracted from peripheral blood leukocytes with the QIAamp DNA Mini Kit (Qiagen, Germany) according to the manufacturer's protocol. WES was performed on genetic DNA from peripheral blood. After genomic DNA fragmentation, paired-end adaptor ligation, amplification, and purification, human exons were captured by using the SepCap EZ Med Exome Enrichment Kit (Roche NimbleGen). The DNA library was obtained by postcapture amplification and purification and then sequenced on the Illumina HiSeq sequencing platform. By using the NextGene V2.3.4 software, sequence data alignment to the human genome reference (hg19) and variant-calling were performed to obtain the coverage and average read depth of the target regions. The mean coverage of the exome was >100×, which enabled us to examine the target region with sufficient depth to accurately match >99% of the target exome. To guarantee the accuracy of data analysis, variants with low coverage were filtered out. Sanger sequencing was used to validate the potential variants discovered by WES, as previously described [17].

To predict the possible pathogenic effects of the novel missense variant, we used different online software tools such as PolyPhen-2 (https://genetics.bwh.harvard.edu/pph2/), PROVEAN (https://provean.jcvi.org/index.php) and Mutation Taster (https://www.varianttaster.org/). Multiple sequence alignment was performed by using Clustal Omega (https://www.ebi.ac.uk/Tools/msa/clustalo/) to confirm the conservation of amino acid substitutions at mutated positions. In addition, a homodimer model of SEMA3A was established by using Swiss-model software (https://www.swissmodel.expasy.org/), and DeepView software (https://www.expasy.org/spdbv/) was applied to observe the effect of this variant on protein structure.

### 2.4. Construction of Plasmids

The full length of the major transcript of human *SEMA3A* (transcript ID: NM_006080.2) was synthesized and cloned into the pcDNA3.1 vector by GeneArt Gene Synthesis (Thermo Fisher Scientific, Rockford, IL). The mutant *SEMA3A* (c.814G > *T*, p.D272Y) was created by the QuickChange Site-Directed Mutagenesis Kit (Stratagene, La Jolla, USA) according to the manufacturer's instructions and then subcloned into pcDNA3.1. The entire coding sequences of all the constructs were verified by sequencing.

### 2.5. Cell Transient Transfection

COS-7 cells were cultured in monolayers in 5% CO_2_ at 37°C in Dulbecco's modified Eagle's medium (Life Technologies, Inc., US) containing 1 mM sodium pyruvate, 2 mM glutamine, 50 mM glucose, and added with 10% fetal bovine serum (Invitrogen, Carlsbad, CA), 100 mg/ml streptomycin, and 100 U/ml penicillin. Then, when COS-7 cells were in good condition, they were transiently transfected with plasmids containing the wild-type *SEMA3A* (pcDNA3.1-*SEMA3A*-WT) and the variant *SEMA3A* (pcDNA3.1-*SEMA3A*-Mu) by using Lipofectamine 3000 transfection reagent (Thermo Fisher Scientific). Cells and their conditioned medium were collected 48 h after transfection to test for the expression of *SEMA3A* by western blot analysis and enzyme linked immunosorbent assay (ELISA).

### 2.6. Western Blot Analysis

Cells were lysed by mammalian protein extraction reagent (ThermoFisher Scientific) and protease inhibitor cocktail (Sigma Aldrich), then mixed with Laemmli buffer containing 2-mercaptoethanol. Proteins were separated by 10% sodium dodecyl sulfate polyacrylamide gel electrophoresis (SDS-PAGE) and transferred onto polyvinylidene fluoride membranes. Then, membranes were incubated overnight with rabbit monoclonal anti*SEMA3A* antibody (1 : 1000, Abcam, Cambridge, UK). After membranes were washed three times for 10 min with 0.1% tris-buffered saline Tween, they were incubated with mouse secondary antibodies for 1 h. Before 5 min of incubation with chemiluminescent HRP substrate, membranes were washed three times again. Finally, bands were visualized by the Alpha Fluorchem *Q* System. Each experiment is performed at least three times.

### 2.7. Enzyme Linked Immunosorbent Assay (ELISA)

The conditioned medium of COS-7 cells was concentrated by the Ultra-3 filter tubes (30 kDa NMWL, Millipore) after 48 h of transfection. The expression of secreted protein *SEMA3A* in culture supernatants was detected by using an enzyme linked immunosorbent assay according to the instructions (Human *SEMA3A* ELISA Kit, Elabscience, Wuhan, China). Then, the optical density (OD) was quantified at a wavelength of 450 nm to calculate protein concentration. Each experiment is performed at least three times.

### 2.8. Statistical Analysis

Statistical analysis was performed by Prism 8.02 (GraphPad Software, San Diego, CA). Each experiment was carried out at least three times independently. A repeated-measure ANOVA followed by Bonferroni post hoc tests or an unpaired two-tail Student's *t*-test was used. *P* < 0.05 was considered statistically significant.

## 3. Results

### 3.1. Clinical Manifestation

The KS pedigree was shown in Figure 2(a). The proband complained of infertility when he first came to our outpatient clinic with his wife. When we asked about his medical history, we found that he had an insensitive sense of smell as well as delayed puberty. Therefore, he took a smell identification test ('Sniffin' Sticks 16-identification test, SIT-16). The result showed that he only recognized 9 types of odors and was assessed as hyposmia. Biochemical testing (shown in Table 1) revealed that testosterone, follicle-stimulating hormone (FSH), and luteinizing hormone (LH) were at a low level and estradiol was slightly elevated. After performing a GnRH stimulation test by triptorelin acetate injection, LH showed a delayed peak with a level of 3.7 mIU/ml at 90 minutes, while normal people have LH peaks between 15 and 45 minutes. The semen routine indicated that the number and motility of sperm were both at low levels, and the specific values are shown in Table 1. No other abnormalities were found in other biochemical examinations. An ultrasonic examination showed his bilateral testicles were of a small size, with a volume of 3.1 × 2.1 × 1.3 cm (left) and 3.2 × 2.2 × 1.4 cm (right). Additionally, he had the development of bilateral mammary glands at Tanner III with a thickness of 1.2 cm (left) and 1.5 cm (right). Kidney ultrasound showed no obvious abnormalities and the chromosome karyotype was 46, XY. Additionally, the father of the proband also has anosmia and has been diagnosed with Kallmann syndrome in another hospital. Surprisingly, he never had any treatment and gave birth to the proband naturally. The siblings of both the proband and the father are normal. All these data indicated the primary diagnosis as KS. And he was given an intramuscular injection of chorionic gonadotropin injection 2000 iu twice a week and oral administration of 40 mg of testosterone undecanoate capsule three times a day.

### 3.2. Variant Analysis

To further confirm the primary diagnosis and explore its genetic causes, whole-exome sequencing was applied to the patient, and Sanger sequencing was used to further confirm the detected variants in all available family members. As a result, we identiced a novel heterozygous variant (NM_006080.2: c.814G > *T*) in the *SEMA3A* gene in the proband and his father, causing the substitution of aspartic acid to tyrosine in codon 272 (p.D272Y Figure 2(b)). The variant was not reported in the literature and was not detected in the HGMD (https://www.hgmd.cf.ac.uk/), gnomAD (https://gnomad.broadinstitute.org), or in a panel of 200 normal Chinese controls. In addition, the pedigree analysis revealed autosomal dominant (AD) inheritance, and the variant segregation within a family is consistent with AD inheritance.

### 3.3. Bioinformatic Analysis

Bioinformatic analysis was used to predict the pathogenicity of this novel variant. It was predicted to be possibly damaging with a high score of 1.000 by using PolyPhen v.2 (Figure 1(a)), and PROVEAN predicted this variant to be deleterious with a score of −8.646, far below the cutoff of −2.5. The Mutation Taster also indicated that the amino acid sequence alteration might affect protein features, which seemed to have a disease-causing effect. Moreover, multiple sequence alignments showed that p.D272Y is highly conserved across various species by using the Clustal X v.2 (https://clustal.org/clustal2/) (Figure 1(b)), revealing that it may have a destructive effect on the structure of the protein.

Notably, the novel c.814G > *T* (p.D272Y) missense variant was located in the sema domain(Figure 1(c)), which may have a key influence on *SEMA3A* signaling [18]. Then, the model of the *SEMA3A* homodimer was built by SWISS-MODEL automatically based on the template and visualized by DeepView. The results showed that the overall spatial structure of the mutant protein D272Y was relatively looser than that of the wild type. In terms of amino acid properties, the original negatively charged acidic state had become the uncharged neutral polarity state, which was the carbonyl group became a benzene ring (Figure 1(d)). Ultimately, it altered the charge distribution of the entire protein and its stability. Therefore, through bioinformatics analysis, we speculate that this novel variant has pathogenic significance.

### 3.4. Functional Analysis

**Figure 3:**
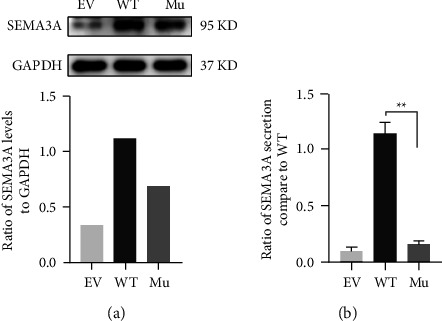
Defective expression of the mutant *SEMA3A* proteins *in vitro*. (a) The intracellular SEMA3A production of COS-7 cells transfected with the pcDNA3.1-SEMA3A-Mu decreased by about 30% compared with that of cells transfected with pcDNA3.1-SEMA3A-WT through western blot. Since SEMA3A was also slightly expressed in kidney cells, the EV group also showed a low level of expression. (b) The ELISA results showed that the protein level of the mutant *SEMA3A* in the condition medium from COS-7 cells was significantly lower than that of the wild-type *SEMA3A* by about 80%. Each experiment was carried out at least three times independently. A repeated-measure ANOVA followed by Bonferroni post hoc tests or unpaired two-tail Student's *t*-test was used. ^*∗∗*^*P* < .01. EV: empty vector; WT: wild-type; Mu: variant.

To explore the pathogenic effect of the novel *SEMA3A* variant at the protein level, plasmids containing the wild-type SEMA3A, mutant *SEMA3A*, and their negative control were respectively transfected into COS-7 cells. As a result, the intracellular SEMA3A production of COS-7 cells transfected with the pcDNA3.1-*SEMA3A*-Mu decreased by about 30% compared with that of cells transfected with pcDNA3.1-*SEMA3A*-WT through western blot (Figure 3(a)). Since SEMA3A was also slightly expressed in kidney cells, the EV group also showed a low level of expression. Meanwhile, ELISA results showed that the level of the mutant SEMA3A in the conditioned medium from COS-7 cells was significantly lower than that of the wild-type SEMA3A by about 80% (Figure 3(b)). All the above results reveal that this novel variant can significantly decrease the expression of SEMA3A and probably affect the functions of *SEMA3A*.

### 3.5. Summary of *SEMA3A* Variants and Its Molecular Pathogenic Mechanism

According to the human gene variant database (HGMD) (https://www.hgmd.org/), all the reported *SEMA3A* variants associated with KS, including our newly identified variant, were listed and summarized in this paper. There were 19 variants of *SEMA3A* related to KS, including 16 missense, 1 splicing, 1 small deletion, and 1 gross deletion (Table 2) [11, 12, 16, 19–21]. 68.4% of the variants are located in the Sema domain, with only 6 variants in non-Sema domains. All of them had recognized gonadal dysplasia and anosmia or hyposmia, one had additional anomalies such as clift lip and dental agenesis, and one had cryptorchidism among the 8 known patients. Notably, 20.8% of the patients had additional variants in two or more genes.

**Figure 4:**
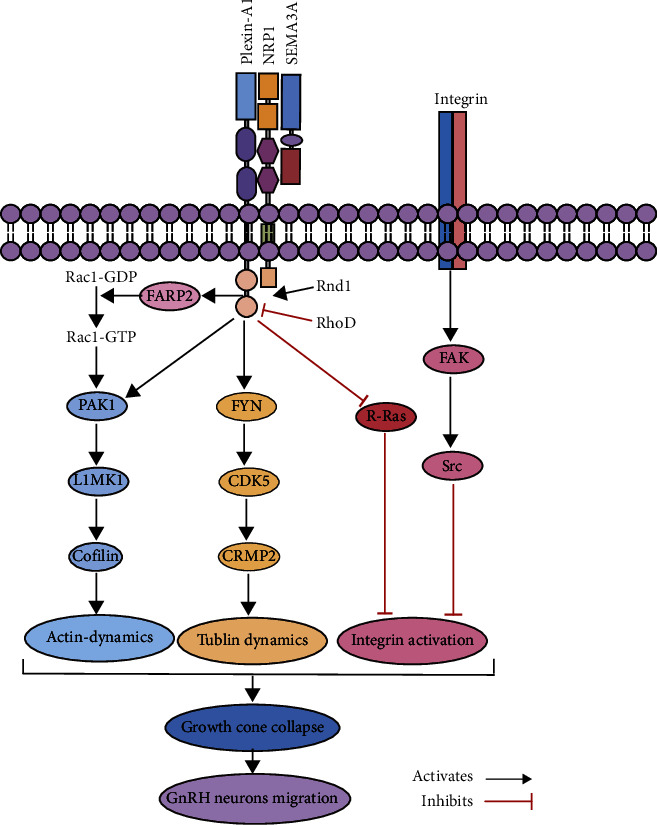
Mechanism of *SEMA3A* signaling. The formation of the SEMA3A-NRP-Plexin complex induces the release of FARP2, which can exchange the small G-protein Rac1-GDP to Rac1-GTP. Then, it sequentially activates p21-activated kinase (PAK), LIM kinase 1 (LIMK1), and the actin-binding factor Cofilin, which finally induces actin dynamics and subsequently shifts the balance of Rnd1 and RhoD activity towards this function. Once activated, Plexin can in turn trigger cytoplasmic tyrosine kinases FYN and the serine/threonine kinase CDK5, as well as the collapsin response mediator protein CRMP2, thereby resulting in tubulin dynamics. Activation of plexin-A1 also leads to inactivation of R-RAS, which regulates the function of integrin, resulting in the inactivation of integrin, thereby promoting the separation of target cells and extracellular matrix. *SEMA3A* can also cause the phosphorylation and dephosphorylation of FAK, thereby activating the downstream Src kinase to achieve the inhibitory effect on integrins. The four pathways mentioned above can eventually cause the growth cone to collapse and ultimately affect the migration of neuronal cells.

Although the molecular mechanism of *SEMA3A* signaling is not clear at present, there are some reported pathways for analysis (Figure 4). To exert its effects, *SEMA3A* binds to a transmembrane coreceptor complex in which neuropilin 1 (NRP1) or NRP2 serves as a signal transducing subunit and PlexinA1 or PlexinA3 as a signal transducing element, resulting in neuronal collapse [14, 22]. The formation of the SEMA3A-NRP-Plexin complex induces the release of FARP2, which can exchange the small G-protein Rac1-GDP to Rac1-GTP. Then, it sequentially activates p21-activated kinase (PAK), LIM kinase 1 (LIMK1), and the actin-binding factor Cofilin, which finally induces actin dynamics and subsequently shifts the balance of Rnd1 and RhoD activity towards this function [23]. Once activated, Plexin can in turn trigger the cytoplasmic tyrosine kinases FYN, the serine/threonine kinase CDK5, and the collapsin response mediator protein CRMP2, thereby resulting in tubulin dynamics [24]. Activation of plexin-A1 also leads to inactivation of R-RAS, which regulates the function of integrin, resulting in the inactivation of integrin, thereby promoting the separation of target cells and extracellular matrix [25]. Additionally, *SEMA3A* can cause the phosphorylation and dephosphorylation of FAK, thereby activating the downstream Src kinase to achieve the inhibitory effect on integrins, which leads to the realization of axon remodeling [26]. The pathways mentioned above can eventually cause the growth cone to collapse and ultimately affect the migration of neuronal cells.

## 4. Discussion

KS is a rare clinically and genetically heterogeneous disease which mainly affects the development of the reproductive system and olfactory organs and eventually leads to infertility and anosmia/hyposmia [27]. The proband in this study had delayed puberty, hyposmia, low levels of sex hormones, small testicular size, infertility, and KS history, which strongly indicated the preliminary clinical diagnosis was KS. Then, we assessed all CHH-related causative genes by WES and identified a novel variant in *SEMA3A* in the proband and his father, which validated the initial diagnosis. To our knowledge, this missense variant has not yet been reported elsewhere. In order to further explore the molecular genetic etiology, we carried out a detailed biological analysis of the variant by using bioinformatics software and verified the expression of it by *in vitro* experiments. Furthermore, a detailed molecular analysis of the variant might provide useful insights into the function of the *SEMA3A*.

SEMA3A is a transmembrane-secreted protein, which is involved in axonal pathfinding during the development of the nervous system [12, 28]. Notably reduced expression of *SEMA3A* has been shown to cause a Kallmann-like phenotype in mice [11]. Our study found that the novel missense variant (p.D272Y) caused an apparent reduction in SEMA3A expression and secretion not only intracellularly but also predominantly outside the cells. Besides, it should be noted that the decrease was more in extracellular than intracellular, proving that the reduction of the extracellular functional protein may be responsible for the disease. Additionally, the variant was predicted to result in deleterious consequences with a high damaging score analyzed by PolyPhen-2, PROVEAN Protein, and Mutation Taster. Co-segregation of the variant and phenotype suggested that it was likely to be pathogenic. The high conservation at position 272 aspartic acid among different species analyzed by Clustal X v.2 suggested that it was structurally and functionally important. Moreover, the novel variant located in the sema domain altered the charge distribution of the entire protein and its stability. All these results show that the novel *SEMA3A* variant was probably harmful and pathogenic in the patients presented here.

Variants in genes affecting the migration of GnRH-specific neurons and the development of the nasal placode are the key pathogenic factors leading to KS [29]. In the 1990s, SEMA3A was first recognized as an evolutionarily conserved axon-guidance cue which contributed to olfactory axon patterning and the migration of GnRH neurons from the nasal placodes to the brain [30]. It has been reported that SEMA3A signaling is dependent on the formation of a 2 : 2 : 2 complex between SEMA3A, PlexinA1, and NRP1 bound to a semaphorin homodimer [31], where the binding of PlexinA1 to NRP1 increases the affinity of SEMA3A to NRP1 [24]. Interestingly, the novel variant occurring both in the putative binding site and in the homodimer interface may affect protein-protein interactions that are associated with functions of the coreceptor complex [13] (Figure 3(a)). Additionally, the homodimer structure is necessary for the high affinity of SEMA3A to the NRP receptor [32].

Furthermore, the axonal growth cone collapse induced by SEMA3A through reorganization of actin filaments, fluid-phase endocytosis, and disruption of integrin-mediated adhesion is vital for cell migration and diffusion [33, 34]. It was observed in the cultured cerebral cortex and hippocampal neurons that FAK downstream of integrin is the central signaling molecule for SEMA3A to induce growth cone collapse [35]. A recent study reported that the missense variant of *SEMA3A* failed to induce FAK phosphorylation in GN11 cells. It is worth noting that the novel variant SEMA3A (p.D272Y) in this study was similar to the above-mentioned locus and had the same variant type, which suggests that p.D272Y may also have this effect [36]. Furthermore, a significant reduction of the variant SEMA3A expression may affect the normal conduction of the other molecular pathways mentioned above.

KS was previously thought to be a monogenic disorder following autosomal dominant, autosomal recessive, and X-linked recessive inheritance, which was later shown to be an oligogenic pattern of inheritance [37]. Oligogenic inheritance has been reported in 10–20% of CHH cases at present [38, 39]. Studies have found that the presence of multiple pathogenic variants may have synergistic effects to produce different degrees of superposition, resulting in relatively severe phenotypes [40]. Compared with KS cases reported previously, the proband had a milder phenotype and no other non-reproductive phenotypic abnormalities except for defects in the sense of smell and reproductive system. Notably, other than the *SEMA3A* variant, no other CHH-related genetic variants were found in the proband, which may be associated with the milder phenotype. However, it seemed inconsistent with the notion that monoallelic mutations in *SEMA3A* were not sufficient to cause abnormal phenotypes in patients reported by Hanchate et al. [12]. But 19 (79.2%) out of the 23 KS patients listed in Table 2 had only a single-gene variant of *SEMA3A*, which was consistent with the traditional view that KS was a monogenic disease, while the remaining had additional variants in two or more genes, which was consistent with the previously reported oligogenic inheritance pattern of KS, revealing the genetic heterogeneity of KS [37, 41]. In addition, variable expression of phenotypes is common in CHH patients, which may be connected to oligogenic inheritance, sex differences, and environmental factors. However, due to the unavailability of patient details and the lack of previous reports of *SEMA3A* variants related to KS, the sample size for analysis was a bit small.

The continuous reversal, including hormone levels, fertility, testicular or penile volumes, of KS and idiopathic hypogonadotropic hypogonadism can be obtained naturally or after discontinuation of therapy in about 10–20% of patients, which suggests the plasticity of the GnRH neuronal network [42]. The proband's father gave birth to the proband without regular treatment, suggesting that the proband may also reverse in the future. However, most cases of IHH require long-term hormone replacement therapy to induce secondary sexual characteristics and maintain sexual function [43]. The therapies of IHH largely depend on the therapeutic goals, such as normal sexual development or reproductive capacity [2]. Remarkably, it is crucial for males during the infancy period to acquire combined gonadotropin therapy to attenuate the psychological effects of small penises in late adolescence and increase fertility in adulthood [9]. Thus, more research needs to be done to provide patients with timely diagnosis and prompt treatment, which are essential to their prognosis.

Undeniably, some limitations existed in our research. The proband with KS refused to undergo a comprehensive examination, such as the olfactory MRI. Additionally, further basic experiments have not been done to specifically identify molecular mechanisms because it was difficult for us to get GN11 cells.

## 5. Conclusion

In summary, we identified a novel missense variant in *SEMA3A* from a Chinese KS pedigree, extending the genetic variant spectrum of *SEMA3A* and providing more data about the heterogeneity of KS. Functional defects of SEMA3A were revealed by bioinformatic analysis and *in vitro* experiments. These results provide additional data for KS patients for genetic counseling, help to identify the function of SEMA3A, and reveal the underlying pathogenesis of KS.

## Figures and Tables

**Table 1:** The results of auxiliary examination on the KS proband.

Clinical examination	Results	Reference range
Height/weight (cm/kg)	175/86	—
Biochemical testing		
FSH (mIU/ml)	2.3	1.5–12.4
LH (mIU/ml)	1.8	1.7–8.6
Testosterone (ng/ml)	1.9	2.8–8.0
Estradiol (pg/ml)	46.64	11.3–43.2
Prolactin (ng/ml)	12.36	4.79–23.3
GnRH stimulation test (mIU/ml)	3.7 (LH peak appeared at 90 min)	—
Semen routine		
Specimen volume (ml)	0.5	≥1.5
Number of sperm (×10 ∧ 6/time)	21.85	≥39
PR + NP (%)	18.10	≥40
PR (%)	9.70	≥32
NP (%)	8.40	—
IM (%)	81.90	—
SIT-16	Hyposmia	—
Ultrasound of the genital system	Small volume of bilateral testis	—
Ultrasound of the breast	Bilateral gynaecomastia	—

KS: Kallmann syndrome; FSH: follicle-stimulating hormone; LH: luteinizing hormone; GnRH: gonadotropin-releasing hormone; SIT-16: sniffing sticks 16-identification test.

**Table 2:** All *SEMA3A* variants reported associated with Kallmann syndrome.

Mutation site	Domain involved	Gender	Inheritance	Gonadal dysplasia	Olfactory state	Additional anomalies	Additional related mutation
c.196C > *T* (p.R66W)	Sema	*M*	*U*	Yes	*A*	No	*PROKR2* p.L218P
		*U*	*U*	Yes	*U*	*U*	No
c.245T > *G* (p.L82R)	Sema	*U*	*U*	Yes	*U*	*U*	*SEMA3A* p.R66W
c.458A > *G* (p.N153S)	Sema	*F*	Inherited	Yes	*U*	Clift lip and dental agenesis	*FGFR1* p.R609^*∗*^
	Sema	2M	*U*	Yes	*U*	*U*	No
c.814G > *T* (p.D272Y)	Sema	*M*	Inherited	Yes	*H*	No	*No*
c.869G > *A* (p.R290H)	Sema	*M*	*U*	Yes	*A*	No	*No*
c.1025T > *C* (p.M342T)	Sema	*U*	*U*	Yes	*U*	*U*	No
c.1198A > *G* (p.I400V)	Sema	*M*	*U*	Yes	*U*	*U*	*PROKR2* p.R268C
c.1253A > *G* (p.N418S)	Sema	*F*	Inherited	Yes	*U*	No	*FGFR1* p.S436Yfs^*∗*^3
c.1340A > *G*(p.D447G)	Sema	*U*	*U*	Yes	*U*	*U*	No
c.1372G > *T* (p.V458I)	Sema	*M*	Inherited	Yes	*U*	Cryptorchidism	No
c.1450C > *T*(p.R484W)	Sema	*U*	*U*	Yes	*U*	*U*	No
c.1971A > *G* (p.I657M)	Ig	*U*	*U*	Yes	*U*	*U*	No
c.2062A > *G* (p.T688A)	Interdomain	*M*	*U*	Yes	*U*	*U*	KAL1 p.Y217D
c.2189G > *A*(p.R730Q)	Basic motif	*F*	*U*	Yes	*U*	*U*	No
		*U*	*U*	Yes	*U*	*U*	No
c.2198G > *A* (p.R733H)	Basic motif	*M*	*U*	Yes	*U*	*U*	No
c.2201G > *A*(p.R734Q)	Basic motif	*U*	*U*	Yes	*U*	*U*	*U*
c.1360 + 2T > *G*	Sema	*U*	*U*	Yes	*U*	*U*	*U*
c.1613_1626del14 (p.Asp538Valfs^*∗*^31)	PSI	*M*	*U*	Yes	*U*	*U*	No
213 kb incl. Ex. 7–17	Sema, PSI, Ig	2M	Inherited	Yes	*H*	No	No
		*F*	Inherited	Yes	*A*	No	No

M: male; F: female; U: unknown; H: hyposmia; A: anosmia.

## Data Availability

The data used to support the findings of this study are available from the corresponding author upon request.
